# Acupuncture and acupressure with improved cancer-related depression of retrospective studies

**DOI:** 10.3389/fonc.2022.1036634

**Published:** 2022-12-12

**Authors:** Feiqing Wang, Jianing Zhao, Yanju Li, Xu Yang, Dan Wu, Bo Yang, Chike Zhang, Zhixu He, Liang Du, Xiaodong Zhu, Dong Ming, Yang Liu, Dongxin Tang

**Affiliations:** ^1^ Clinical Medical Research Center, The First Affiliated Hospital of Guizhou University of Traditional Chinese Medicine, Guiyang, Guizhou, China; ^2^ Academy of Medical Engineering and Translational Medicine, Tianjin University, Tianjin, China; ^3^ Department of Hematology Oncology, Affiliated Hospital of Guizhou Medical University, Guiyang, Guizhou, China; ^4^ Key Laboratory of Adult Stem Cell Translational Research, Chinese Academy of Medical Sciences, Guizhou Medical University, Guiyang, Guizhou, China; ^5^ Chinese Evidence-Based Medicine Center, West China Hospital, Sichuan University, Chengdu, China; ^6^ Neurological Institute, Tianjin Medical University General Hospital, Tianjin, China

**Keywords:** acupuncture, acupressure, acupoints, cancer/tumor/oncology, depression

## Abstract

**Introduction:**

Acupuncture and acupressure are widely used for treating cancer pain and depression and recognized as safe and effective by the international medical community. In this study, we systematically evaluated the efficacy, safety, and clinical significance of acupuncture and acupressure in treating cancer-related depression.

**Methods:**

We searched MEDLINE, PubMed, Science Direct, Google Scholar, Web of Science and Embase and Chinese-language databases for randomized clinical trials (RCTs). To assess efficacy, rating scales administered by clinicians or experts were preferred, including the Hamilton Depression Rating Scale (HAMD), Self-rating Depression Scale (SDS), Self-rating Anxiety Scale (SAS), and Quality of Life Questionnaire-Core 30 (QLQ-C30) and the total effective rate after treatment. In all, Sixteen RCTs involving 1019 cancer patients were included in the Meta-analysis.

**Results:**

Eleven (69%) of these studies reported the post-treatment total effective rate. Three hundred fifty-three patients received antidepressants; the total effective rate was 72.5%. Three hundred sixty-one patients underwent acupuncture and acupressure; the total effective rate was 90%. Meta-analysis results showed I^2^ = 0%, no heterogeneity, (Z = 5.84, *p* < 0.00001); and combined OR = 3.55, (95% CI = 2.32 to 5.43).

**Discussion:**

This study found that acupuncture and acupressure are as effective as medication in the treatment of cancer-related depression, provide a reliable basis for the clinical use of acupuncture to treat cancer-related depression, help promote nonpharmacological treatment for cancer-related complications. These approaches thus help reduce drug resistance and adverse reactions and improve patients’ quality of life.

## Introduction

According to the report “2018 Global Cancer Statistics” published by the official journal of the American Cancer Society, there were 18.1 million new cancer cases worldwide (1, 2), the number of new cases in China was 3.804 million, an average of 10,000 people are diagnosed with cancer every day (3), and the severe cancer burden poses a severe challenge to global public health. Cancer not only causes the development of obvious physical lesions but also impacts the psychological bottom line of patients. This bad mood not only reduces the quality of life of patients but also severely shortens their life cycle. The most common negative emotions in patients with cancer are anxiety and depression (4). The symptoms are mainly low mood, loss of interest, lack of energy, lack of physical strength, pessimism, guilt, and suicidal tendencies, but not psychotic depression (5, 6). Therefore, psychological diseases caused by cancer have attracted greater attention from the medical field.

Cancer-related depression, also known as tumor depression, is a type of emotional pathological reaction. This depression is caused by a cancer diagnosis, treatment, and complications, and the incidence rate is as high as 58% (7). A psychiatric research team led by Jane Walker, MD, University of Oxford, UK, recently analyzed 21,151 cancer patients from several clinics in Scotland and found that the prevalence of depression in cancer patients is markedly higher than that in the general population, with the prevalence being the highest among lung cancer patients (8). However, depression is likely to be the main cause for malignant tumor death (9), with estimates as high as a 26% higher mortality rate among patients with depressive symptoms and a 39% higher mortality rate among those with a diagnosis of major depression (10). Cancer depression not only reduces the quality of life of patients but also shortens the patients’ life in severe cases. Therefore, clinical treatment is not only necessary to treat the primary disease but also to alleviate patients’ depression and improve their quality of life, which is also important for delaying survival. At present, antidepressants are still the main treatment for tumor depression. However, antidepressants have a slow onset, short-term effects, easy tolerability, many side effects, and an adverse drug reaction prevalence of 31-60%. Furthermore, they are not helpful for the management of nausea and vomiting and hypofunction of the spleen and stomach after radiotherapy and chemotherapy (11).

Acupuncture and acupressure, both of which are traditional therapies, have been widely used in the treatment of cancer pain and mental depression, and their clinical safety and efficacy have been confirmed (12, 13). The reason acupuncture and acupressure have received attention and recognition at home and abroad and become among the methods recommended by the World Health Organization (WHO) for the treatment of depression is that they afford advantages including high safety, few adverse reactions, rapid onset of treatment effects, good long-term effects, and ease of acceptance by patients (14, 15). According to the WHO, acupuncture and acupressure are used in at least 183 countries. Furthermore, 18 countries and regions have included it in their national health insurance system, and acupuncture and massage have become the most widely used traditional medical therapies in the world (16). In the United States, acupuncture and acupressure can be considered for improving mood disturbance and depressive disorder, and many tumor medical institutions now offer acupuncture and acupressure treatment technology (17). Furthermore, international cancer organizations such as National Comprehensive Cancer Network (NCCN) highly recommend nonpharmacological interventions, acupuncture and acupressure for treated cancer pain (18). In countries including the United Kingdom, Germany, and Switzerland, acupuncture and acupressure have been available of the National Health Service (NHS) for decades as a nonpharmacological intervention for the management of acute or chronic pain (19).

In light of the growing number of randomized clinical trials (RCTs) of acupuncture and acupressure use for the management of cancer-related depression, the purpose of this study was to identify prospective RCTs investigating the use of acupuncture and acupressure for application effect and value among patients with cancer, provide useful information for clinicians regarding use of acupuncture and acupressure in patients with cancer-related depression.

## Materials and methods

### Search strategy and selection criteria

English databases (MEDLINE, PubMed, Science Direct, Google Scholar, Web of Science and Embase) and Chinese databases (CNKI, Wanfang Database, and Chinese Biomedical Literature Database) were searched for RCTs published from database inception through June 30, 2022. The search strategy consisted of three components: clinical condition (tumor/tumour, carcinoma, cancer, oncology, neoplasm and depression, and antidepressants), intervention (acupuncture and moxibustion, hand acupuncture, electro-acupuncture, body or auricular acupressure), and study type RCTs.

### Data collection process

Three of the authors (WFQ, ZJN and LYJ) independently performed the data extraction, to establish the eligibility of the studies. Studies published in Chinese or English databases were included if they were RCTs investigating the association of acupuncture and acupressure with cancer-related depression. Studies on depression directly accompanying the development of cancer and/or depression associated with cancer chemotherapy were included. The eligible interventions were acupuncture and acupressure. Clinical features includes journal published, year, source, participants, interventions, treatment details, methodological characteristics, and the results for each outcome were extracted for each study. The comparison could be between selective serotonin reuptake inhibitors (SSRIs) and antidepressant therapy. Studies comparing acupuncture and acupressure with other traditional Chinese medicine therapies (e.g., herbal medicine) were excluded. The degree of depression, negativity, insomnia and agitation was selected as the targeted outcome because of its substantial role in related depression assessment.

To assess efficacy, rating scales administered by clinicians or experts were preferred, including the Hamilton Depression Rating Scale (HAMD) (20), Self-rating Anxiety Scale (SAS) (21), Self-rating Depression Scale (SDS) (22), and Quality of Life Questionnaire-Core 30 (QLQ-C30) (23), Patient self-rated functional scale (PSFS), Karnofsky Performance Status Scale (KPS) and the total effective rate after treatment.

The HAMD was designed to be administered by researchers and is used to evaluate mood, suicide ideation, anxiety, insomnia, feelings of guilt, retardation. The scale has been widely applied to assess the severity of depression. The quality-of-life questionnaire core 30 (QLQ-C30) was designed by the European Organization for Research and Treatment of Cancer in 1993. It is specially used to assess the quality of life (QOL) of cancer patients. The physiological characteristics of the QLQ-C30 are very good, and its sensitivity, effectiveness, and reliability are very high. It has been used for the QOL measurement of cancer patients in many countries and regions.

### Statistical analysis

This study used the Revman 5.3 statistical software provided by the Cochrane Collaboration Network. The chi-square test (χ^2^) was used to evaluate heterogeneity among the studies (*P* = 0.05). We used a fixed effects model when the heterogeneity was *P* > 0.05. Random effects model for the analysis when heterogeneity was P ≤ 0.05. Weighted mean difference (WMD) was used for continuous variables and Odds ratio (OR) was used for binary variables. Dichotomous and continuous variables were expressed using 95% confidence intervals. The corresponding *P*-value was obtained using the Z test for the combined statistics. Statistical significance was set at *P* ≤ 0.05.

## Results

### Literature search results and study

**Figure 1:**
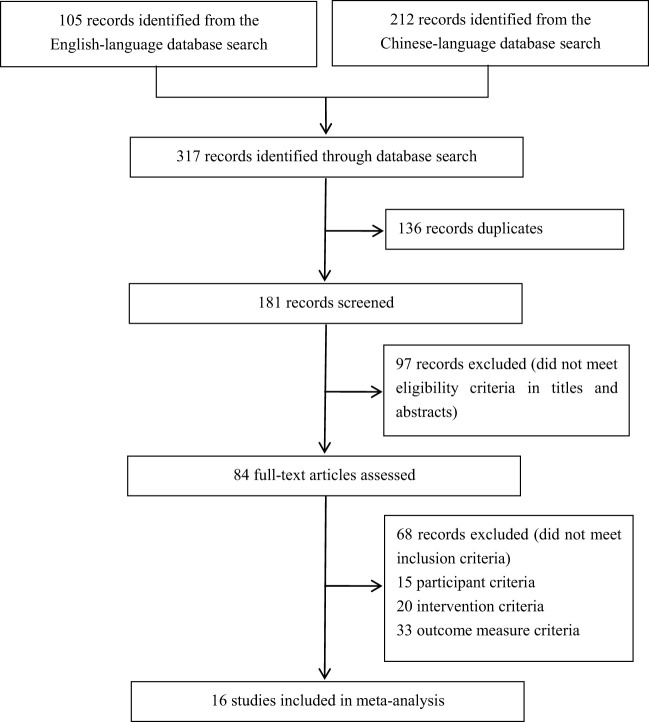
Flow diagram of literature search and study selection.

Out of a total of 317 studies that were retrieved, after application of the exclusion criteria, a total of 16 studies were finally included in the meta-analysis. Sixteen (5%) were included in the statistical analysis (24–39) (**Figure 1**), and the study characteristics of these articalies are summarized in **Table 1**. Among the 16 RCTs included, 14 studies (88%) employed had a parallel-group design, 9 studies compared acupuncture and acupressure with antidepressants (23–26, 28–33), and 5 studies compared the combination of acupuncture and acupressure with analgesic drug treated (35–39).

**Table 1:** Characteristics of the included randomised controlled trial studies for Cancer-Related Depression.

Name	Experimental	Control	Cancer type	Tumor grades		Sample size(Exp/Con)	Age		Gender		Acupoint	Treatment cycle
				ExpⅠ/Ⅱ/III/<	ConⅠ/Ⅱ/III/<		Exp	Con	Exp(male/female)	Con(male/female)		
Pei (2010) (24)	Acupuncture and acupressure	Citalopram hydrobromide (SSRIs)	Breast cancer	NA	NA	31/36	51.76 ± 10.21	48.34 ± 8.79	0/31	0/36	Acupuncture: Feishu, Xinshu, Geshu, Ganshu, Pishu, Shenshu	6 weeks
Feng (2011) (25)	Acupuncture and acupressure	Fluoxetine hydrochloride (SSRIs)	Pulmonary cancer, Gastric cancer, Breast cancer, et al	NA	NA	40/40	63.80 ± 5.47	63.60 ± 4.26	26/14	27/13	Fenglong, Yinlingquan, Xuehai, Sanyinjiao, Yintang, Baihui	30 days
Xiao (2014) (26)	Acupuncture and acupressure	Fluoxetine hydrochloride (SSRIs)	Breast cancer	NA	NA	30/30	52 ± 5	51 ± 5	0/30	0/30	Acupuncture: Taichong, Hegu, Baihui, Zusanli, et al	8 weeks
Lv (2015) (27)	Acupuncture and acupressure	Flupentixol Melitracen	Breast cancer	17/11/2/0	15/14/1/0	30/30	42 ± 5	43 ± 6	NA	NA	Auricular points: Shenmen, Kidney, Liver, Heart, et al	4 weeks
Zeng (2015) (28)	Acupuncture and acupressure	Sertraline hydrochloride (SSRIs)	Lung cancer, gastric cancer, nasopharyngeal carcinoma, rectal cancer, et al	2/11/15/1	1/14/12/1	29/28	60.28 ± 10.23	61.71 ± 11.58	12/17	13/15	Acupuncture: Neiguan, Shenmen, Laogong, the Four spirit points, et al	4 weeks
Liu (2016) (29)	Acupuncture and acupressure	Alprazolam	Malignant tumor	NA	NA	20/20	55.9 ± 2.4	56.2 ± 2.5	11/9	10/10	Acupuncture: Fengchi, Shenmen, Baihui,Touwei, et al	25 days
Wang (2017) (30)	Acupuncture and acupressure	Escitalopram oxalate (SSRIs)	Cervical cancer, endometrial cancer, ovarian cancer	NA	NA	39/39	52.95 ± 7.85	53.64 ± 7.67	NA	NA	Shangxig, Shenmai, Fenglong, Sanyinjiao, Ganshu	10 weeks
Xia (2017) (31)	Acupuncture and acupressure	Fluoxetine hydrochloride (SSRIs)	Cervical cancer, endometrial cancer, ovarian cancer Lung cancer, liver cancer, et al	NA	NA	23/23	NA	NA	NA	NA	Scalp Acupuncture : Middle Line of Vertex (MS5), et al	4 weeks
Deng (2018) (32)	Acupuncture and acupressure	Sertraline hydrochloride (SSRIs)	Gynecological malignancies (cervical cancer, fallopian tube tumor,et al	0/0/20/10	0/0/22/8	30/30	48.93 ± 4.03	50.13 ± 4.09	12/18	16/14	Acupuncture: Hegu, Neiguan, Taichong, Yanglingqan, et al	4 weeks
Chen (2018) (33)	Acupuncture and acupressure	Paroxetine hydrochloride (SSRIs)	Gastric cancer, liver cancer, lung cancer, colon cancer, esophageal cancer	NA	NA	18/18	66.34 ± 5.43	65.13 ± 5.98	10/8	9/9	Acupuncture: Neiguan, Shenmen,Yifeng,Baihui, et al	6 weeks
Li (2019) (34)	Acupuncture and acupressure	Paroxetine hydrochloride (SSRIs)	Breast cancer	23/8/1/0	21/6/1/0	32/28	52.37 ± 8.19	51.75 ± 9.27	NA	NA	Acupuncture: Baihui, Yintang, Guanyuan, Qihai, Neiguan, et al	8 weeks
Han (2017) (35)	Acupuncture and acupressure	Flupentixol Melitracen	Lung cancer,digestive system, gynecology, urinary system, et al	2/8/11/27	1/6/10/30	48/47	60.4 ± 8.29	56.51 ± 10.79	21/27	20/27	Acupuncture: Neiguan Auricular points: Heart, et al	6 weeks
Wang (2018) (36)	Acupuncture and acupressure	Fluoxetine hydrochloride (SSRIs)	Oophoroma	NA	NA	40/40	45.7	46.1	21/19	22/18	Acupoint: Liver, Gallbladder, Heart, kidney, Shenmen, et al	4 weeks
Deng (2019) (37)	Acupuncture and acupressure	Sertraline hydrochloride (SSRIs)	Chest tumor, breast cancer, digestive tract tumor, gynecologic tumor	NA	NA	30/30	52.7 ± 8.6	49.4 ± 10.8	11/19	10/20	Acupuncture: Hegu, Taichong, Neiguan, Shenmen, Zusanli, et al	4 weeks
Yan (2019) (38)	Acupuncture and acupressure	Sertraline hydrochloride (SSRIs)	Malignant tumor	NA	NA	30/30	54.5 ± 23.5	54.0 ± 23.0	15/15	14/16	Acupuncture: Hegu, Taichong, Baihui, Yintang, Guanyuan, Qihai	NA
Liu (2019) (39)	Acupuncture and acupressure	Sertraline hydrochloride (SSRIs)	Malignant tumor	0/21/19/0	0/24/16/0	40/40	63 ± 13	63 ± 12	24/16	20/20	Acupuncture: Taichong, Hegu, Baihui, Yintang	3 months

NA, not applicable; SSRIs, Selective Serotonin Reuptake Inhibitor; Exp=Experimental, Acupuncture and acupressure; Con=Control, Selective Serotonin Reuptake Inhibitor.

These 16 studies involved 1019 patients with cancer, 510 (50%) in the experimental group and 509 (50%) in the control group. The distribution of tumors in the 1019 cases was as follows: gastrointestinal tumors (gastric cancer, colorectal cancer, esophageal cancer, anal cancer), 115 patients; gynecological tumors (cervical cancer, fallopian tube tumor, endometrial cancer, ovarian cancer), 185 patients; lung cancer, 83 patients; lymphoma, 14 patients; breast cancer, 286 patients; nasopharyngeal carcinoma, 6 patients; liver cancer, 24 patients; pancreatic cancer, 2 patients; testicular cancer, 4 patients; prostate cancer, 9 patients; thyroid cancer, 12 patients; urinary system (renal cancer), 81 patients; and other cancers, 198 patients. In the literature we have included, the most common international code of acupoints acupuncture points used in the treatment group are Fenglong (ST 40), Yinlingquan (SP 9), Xuehai (SP 10), Sanyinjiao (SP 6), Yintang (EX-HN3), Baihui (DU 20), Sishencong (EX-HN1), Neiguan (PC 6) and Shenmen (TF 4).

### Total effective rate

**Figure 2:**
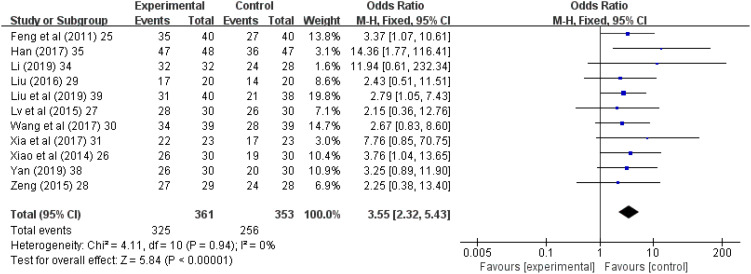
Forest chart of total effective rate after treatment. CI, Confidence Interval; M-H, Mantel Haenszel.

**Figure 3:**
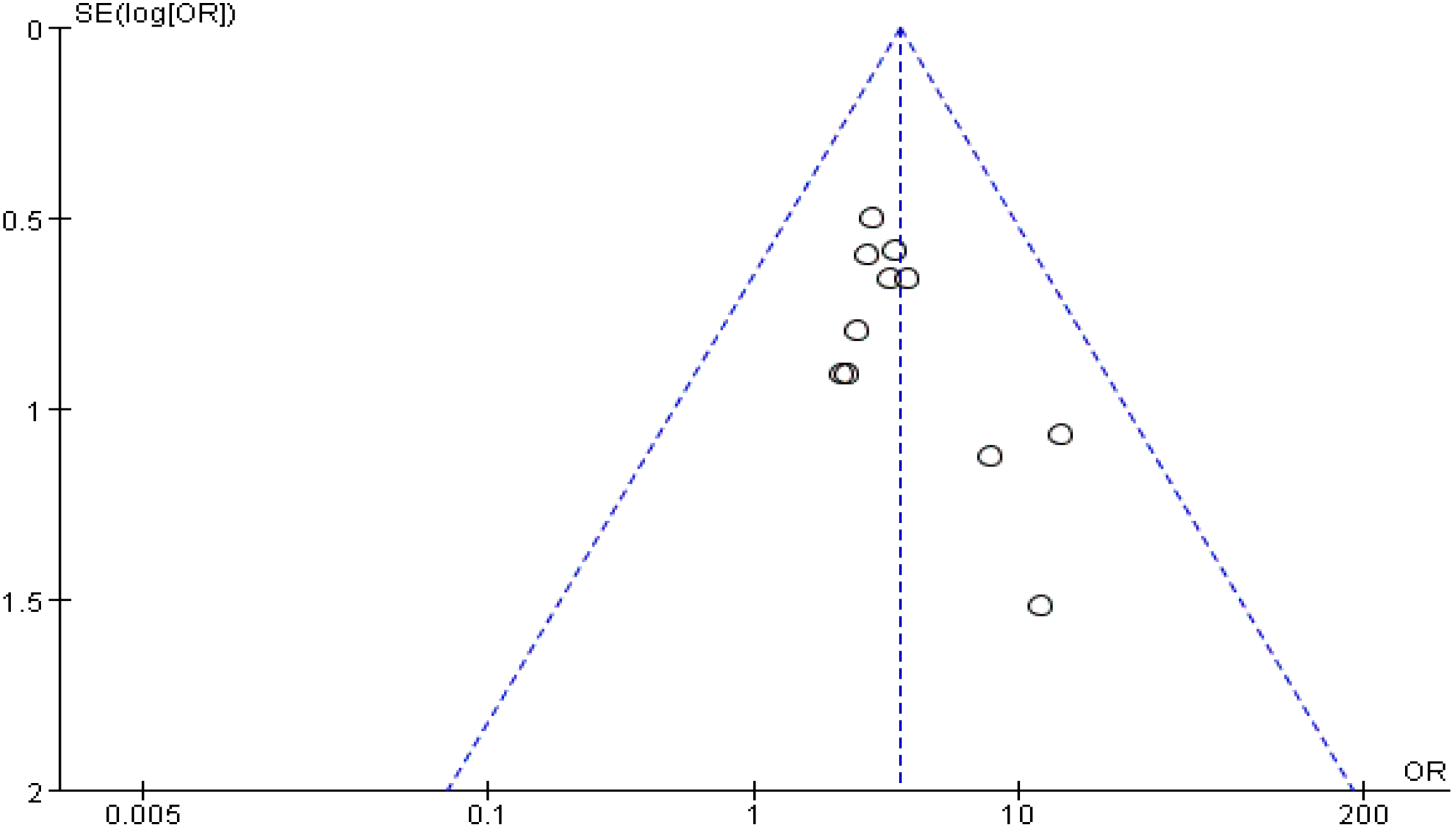
Funnel plot of total effective rate after treatment.

Eleven (69%) (25–31, 34, 35, 38, 39) of the 16 studies reported the total effectiveness rate after treatment. A total of 353 patients were treated with antidepressants, and the total effectiveness rate was 72.5% (256 patients). Among the 361 patients treated with acupuncture and acupressure, the total effectiveness rate was 90% (325 patients). Meta-analysis results showed that I^2^ = 0% with no heterogeneity, so a meta-analysis was performed with a fixed-effects model, and it showed (Z = 5.84, *p* < 0.00001), and combined OR = 3.55, (95% CI = 2.32 to 5.43). This study indicates that acupuncture and acupressure are as effective as medication in treating cancer-related depression (*p* ≤ 0.00001, **Figure 2**). The sensitivity analysis showed that there was no bias in the results (**Figure 3**).

### Hamilton rating scale for depression

**Figure 4:**
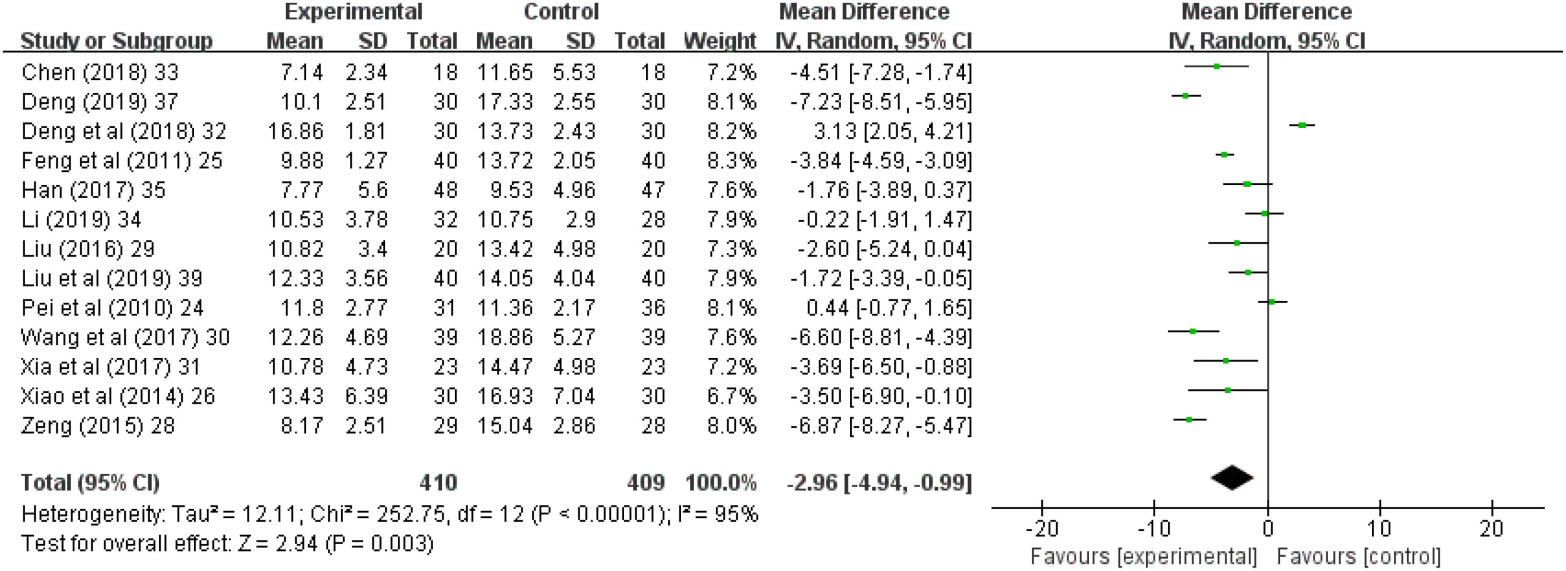
Forest chart of Hamilton Rating Scale for Depression. CI, Confidence Interval; IV, Information Value.

Thirteen (81.3%) (24–26, 28–35, 37, 39) of the 16 studies reported HAMD scores; in these studies, 409 patients were treated with antidepressants and 410 patients were treated with acupuncture and acupressure. The results showed I^2^ = 95% with high heterogeneity, performed with the random-effects model, and it showed (Z = 2.94, *p* = 0.003), and combined WMD = -2.96, (95% CI = -4.94 to -0.99). Thus, acupuncture and acupressure are as effective as medication (*p* ≤ 0.05, **Figure 4**).

### Self-rating depression scale

**Figure 5:**
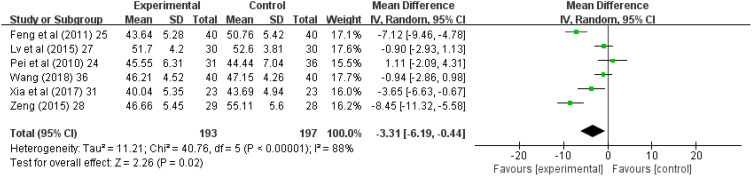
Forest chart of Self-rating depression scale. CI, Confidence Interval; IV, Information Value.

Six (37.5%) (24, 25, 27, 28, 31, 36) of the 16 studies reported SDS scores; in these studies, 197 patients were treated with antidepressants and 193 patients were treated with acupuncture and acupressure. The results showed I^2^ = 88% and high heterogeneity, so a meta-analysis was performed with a random-effects model, and it showed (Z = 2.26, *p* = 0.02); and combined WMD = -3.31, (95% CI = -6.19 to -0.44). Thus, acupuncture and acupressure are as effective as medication (**Figure 5**).

### Quality of life questionnaire-core 30 scale

Eight (50%) (24, 25, 28, 29, 32, 35–37) of the 16 studies reported Quality of Life Questionnaire-Core 30 (QLQ-C30) scores; in these studies, 271 patients were treated with antidepressants and 268 patients were treated with acupuncture and acupressure. The SAS and QOL results showed moderate heterogeneity, so a meta-analysis was performed with a random effects model, and it yielded the following findings: SAS: I^2^ = 68%, (Z = 2.11, *P* = 0.03); and combined WMD = -3.43, (95% CI = -6.60 to -0.25). PSQI: I^2^ = 0%, (Z = 10.66, *p* < 0.00001); and combined WMD = -3.53, (95% CI = -4.18 to -2.88). QOL: I^2^ = 54%, (Z = 4.62, *p* < 0.00001); and combined WMD = 10.76, (95% CI = -6.19 to -15.32). Thus, acupuncture and acupressure are as effective as medication and significantly improved the SAS, PSQI, and QOL of cancer patients (**Table 2**).

**Table 2:** Quality of Life Questionnaire-Core 30 scale.

Project	Test for heterogeneity		Analysis model	Test for overall effect		Mean Difference,IV, 95% CI	Sample size
	*I* ^2^ (%)	*p*-Value		*Z*	*p*-Value		(Exp/Con)
SAS	68%	0.08	Random	2.11	0.003	-3.43 (-6.60 to -0.25)	71/76
PSQI	0%	0.98	Random	10.66	<0.00001	-3.53 (-4.18 to -2.88)	108/107
QOL	54%	0.12	Random	4.62	<0.00001	10.76 (6.19 to 15.32)	89/88

SAS, Self-rating Anxiety Scale; PSQI, Pittsburgh Sleep Quality Index; QOL, the quality of life; CI, Confidence Interval; IV, Information Value; Exp, Experimental; Acupuncture and acupressure; Con, Control; Selective Serotonin Reuptake Inhibitor.

### Patient self-rated functional scale

Patient self-rated functional scale (PSFS) results showed low heterogeneity, so a meta-analysis was performed with a fixed-effects model. The Role Physical (Z = 2.78, *p* = 0.005), Body Function (Z = 5.16, *p* < 0.00001), Cognitive Function (Z = 2.46, *p* = 0.01), Emotional Function (Z = 4.45, *p* < 0.00001), and Social Function (Z = 2.07, *p* = 0.04) of tumor patients showed significant improvements, and acupuncture and acupressure are as effective as medication (*p* ≤ 0.05, **Table 3**).

**Table 3:** Patient self-rated functional scale^*^.

Project	Test for heterogeneity		Analysis model	Test for overall effect		Mean Difference,IV, 95% CI	Sample size
	*I* ^2^ (%)	*p*-Value		*Z*	*p*-Value		(Exp/Con)
Role Physical	8%	0.34	Fixed	2.78	0.005	5.56 (1.64 to 9.47)	89/88
Body Function	84%	0.002	Fixed	5.16	<0.00001	11.47 (7.11 to 15.82)	89/88
Cognitive Function	2%	0.36	Fixed	2.46	0.01	5.78 (1.17 to 10.40)	89/88
Emotional Function	4%	0.31	Fixed	4.45	<0.00001	8.85 (4.95 to 12.76)	59/58
Social Function	0%	0.98	Fixed	2.07	0.04	6.01 (0.32 to 11.69)	59/58

^*^Patient self-rated functional scale includes Role Physical, Body Function, Cognitive Function, Emotional Function and Social Function; CI, Confidence Interval; IV, Information Value; Exp=Experimental, Acupuncture and acupressure; Con, Control; Selective Serotonin Reuptake Inhibitor.

### Karnofsky performance status scale

Karnofsky Performance Status Scale (KPS) results showed moderate heterogeneity, so a meta-analysis was performed with a random effects model. Fatigue (Z = 2.03, *p* = 0.04), Loss of appetite (Z = 3.31, *p* = 0.0009), Ache (Z = 2.38, *p* = 0.02), Dyspnea (Z = 1.94, *p* = 0.05), Diarrhea (Z = 5.31, *p* < 0.00001), Insomnia (Z = 4.97, *p* < 0.00001), Constipation (Z = 1.46, *p* = 0.15), Nausea and vomiting (Z = 1.49, *p* = 0.14) of cancer patients showed significant improvements, and acupuncture and acupuncture and acupressure are as effective as medication (*p* ≤ 0.05, **Table 4**).

**Table 4:** Karnofsky performance tatus scale^*^.

Project	Test for heterogeneity		Analysis model	Test for overall effect		Mean Difference,IV, 95% CI	Sample size
	*I* ^2^ (%)	*p*-Value		*Z*	*p*-Value		(Exp/Con)
Fatigue	88%	0.004	Random	2.03	0.04	-15.29 (-30.08 to -0.51)	59/58
Loss of Appetite	55%	0.14	Random	3.31	0.0009	-16.57 (-26.39 to -6.76)	59/58
Ache	NA	NA	Random	2.38	0.02	-8.83 (-16.10 to -1.56)	29/28
Dyspnea	NA	NA	Random	1.94	0.05	-5.11 (-10.28 to 0.06)	29/28
Diarrhea	NA	NA	Random	5.31	<.00001	-9.19 (-12.58 to -5.80)	29/28
Insomnia	NA	NA	Random	4.97	<.00001	-27.78 (-38.73 to -16.83)	30/30
Constipation	0%	0.57	Random	1.46	0.15	-4.80 (-11.26 to 1.66)	59/58
Nausea and Vomiting	54%	0.14	Random	1.49	0.14	-7.68 (-17.75 to 2.40)	59/58

^*^Karnofsky performance status scale includes fatigue, loss of appetite, ache, dyspnea, diarrhea, insomnia, constipation, nausea and vomiting; CI, Confidence Interval; IV, Information Value; Exp=Experimental, Acupuncture and acupressure; Con, Control; Selective Serotonin Reuptake Inhibitor.

### Safety

The adverse events reported were slight pain from the application of treatment to the skin, or headache and dizziness. The patient’s symptoms were slight, without intervention. Study indicated with a moderate level of certainty that acupuncture and acupressure were associated with reduced cancer-related depression in comparison with antidepressant therapy. The overall effectiveness rate also showed that acupuncture and acupressure reduced anxiety and depression in cancer patients.

## Discussion

The ongoing sedative drugs crisis in the America has exacerbated the challenges surrounding cancer treatment (40–43), with government organizations calling for the use of nonpharmacological interventions (44–46). Acupuncture and acupressure have been widely used in nonpharmacological interventions for cancer pain and various factors causing depression (12, 47–50). Acupuncture and acupressure for the treatment of pain and depression have been shown to be minimally invasive, safe, and associated with minimal adverse effects (17, 51–53). Acupuncture and acupressure are age-old techniques derived from ancient Chinese science, and while they form the essence of Traditional Chinese Medicine. Acupuncture and acupressure, based on the ancient Chinese concept of meridians and the points, they became part of modern medicine in the 1970s (54, 55). In November 2010, UNESCO listed acupuncture and moxibustion on the list of representative works of human intangible cultural heritage. The unique identity of acupuncture lies in the placement of steel needles into specific points on the body that have changes the body’s electrophysiological response (56, 57). Both the NCI and the Cochrarane have sponsored several clinical trials evaluating the use of acupuncture and acupressure for symptom management of cancerous complications, which have shown these techniques to be effective (18). In the NHS, acupuncture and acupressure are administered by physicians or Allied Health Professionals, including “traditional” acupuncturists, sports therapists, osteopaths, and chiropractors (19, 58). Presently, NICE recommends acupuncture and acupressure as a prophylactic treatment for chronic tension-type brain dysfunction (59). This study systematically analyzed the clinical reports of acupuncture and acupressure to improve cancer-related depression.

In patients with cancer, depression and other psychiatric comorbidities are responsible for worsened quality of life (60), lower compliance with anti-cancer treatment (61, 62), extreme behavior (63), and greater psychological burden on the family (64). In our study, the total effectiveness rate in the 353 patients treated with antidepressants was 72.5% (256 patients), while the corresponding rate in the 361 patients treated with acupuncture and acupressure was 90% (325 patients). Thus, acupuncture and acupressure treatment yielded a 17.5% higher effectiveness rate in comparison with drug treatment, and nonpharmacological interventions were significantly better than drug treatment. The results of this study provide an effective basis for the future clinical use of acupuncture and acupressure nonpharmacological interventions in cancer-related depression.

The HAMD was compiled by Hamilton in 1960, and in the absence of another authoritative scale to replace it, it remains the most commonly used scale for clinical evaluation of depression. In our study, HAMD assessments were performed for 409 patients treated with antidepressants and 410 patients treated with acupuncture and acupressure, and acupuncture and acupressure are as effective as medication. SDS was compiled by Professor William W.K. Zung of Duke University and has been recommended by the US Department of Education, Health and Welfare for psychopharmacology research. SDS can accurately reflect the subjective feelings of patients, and is easier to understand than the names of symptoms. In our study, 157 patients treated with antidepressants and 153 patients treated with acupuncture and acupressure were evaluated using SDS, and acupuncture and acupressure are as effective as medication.

QLQ-C30 has been developed by the European Organization for Research and Treatment of Cancer, and is specially designed for cancer patients. It has good specificity, high sensitivity, validity, and reliability, and is easy to understand and complete. Therefore, the questionnaire often yields good compliance and has been widely used (65). In our review, QLQ-C30 assessments were performed for 271 patients treated with antidepressants and 268 patients treated with acupuncture and acupressure. Acupuncture and acupressure are as effective as medication, with significant improvements in SAS, PSQI and QOL of tumor patients. The PSFS results showed significant improvements in Role Physical, Body Function, Cognitive Function, Emotional Function, and Social Function of cancer patients. KPS results showed significant improvements in Fatigue, Loss of Appetite, Ache, Dyspnea, Diarrhea, and Insomnia of cancer patients, and acupuncture and acupressure are as effective as medication. Consensus panel reported that acupuncture and acupressure was effective in controlling cancer pain, chemotherapy-related nausea and vomiting, and mental depression (66). In oncology, complementary and integrative therapies have been employed with the intent of enhancing wellness, improving QOL, and relieving the symptoms of disease and the adverse effects of conventional treatments.

Our study has some limitations. First, we used a standard prescription to evaluate the efficacy of adjunctive acupuncture and acupressure. Second, another limitation of the study is that the evaluation of physical conditions was not pursued in an entirely consistent manner across trials. Third, The experimental data we have included were obtained from various well-known Chinese medicine universities, Chinese medicine research institutes, and well-known Chinese medicine hospitals in China. Acupuncture and acupressure is an ancient Chinese medical technique and has been proficiently used to treat various diseases, and a large amount of credible data on this technique has been accumulated. Moreover, the data sources and statistical methods included in this study are internationally accepted and recognized, and acupuncture and acupressure treatment of cancer-related complications has been used globally, so our research results have important guiding value for the clinical treatment of cancer-related depression. Finally, In most of these studies, baseline antidepressants use was not specified. Consequently, variations in depression type and dose among participants within each study and between studies also likely contributed to heterogeneity.

## Conclusions

With the increasing incidence of tumors and the transformation of the biomedical model to the bio-psycho-social medicine model, this meta-analysis systematically reviewed the effect of acupuncture, acupressure and drugs on cancer-related depression, and the results showed that the effect of acupuncture is as effective as drug treatment, and acupuncture can serve as a supplement to comprehensive treatment of tumors with a multidisciplinary, multimodal, stepwise approach that combines pharmacological interventions with nonpharmacological treatments.

## Data availability statement

The original contributions presented in the study are included in the article/supplementary material. Further inquiries can be directed to the corresponding authors.

## Author contributions

FW, JZ, YLi and YLiu conceived of and designed the study; they had full access to all data in the study and take responsibility for the integrity of the data and the accuracy of the data analysis. FW and YLiu wrote the report. DT, XZ, DM and ZH critically revised the report. JZ, CZ, DW, BY, XY and LD performed the statistical analysis. All authors contributed to the article and approved the submitted version.
